# Reproducibility in Radiomics: A Comparison of Feature Extraction Methods and Two Independent Datasets

**DOI:** 10.3390/app13127291

**Published:** 2024-02-20

**Authors:** Hannah Mary T. Thomas, Helen Y. C. Wang, Amal Joseph Varghese, Ellen M. Donovan, Chris P. South, Helen Saxby, Andrew Nisbet, Vineet Prakash, Balu Krishna Sasidharan, Simon Pradeep Pavamani, Devakumar Devadhas, Manu Mathew, Rajesh Gunasingam Isiah, Philip M. Evans

**Affiliations:** 1 Department of Radiation Oncology, Christian Medical College Vellore, Vellore 632004, Tamil Nadu, India; 2 Centre for Vision, Speech and Signal Processing, University of Surrey, Guildford GU2 7XH, UK; 3 Department of Medical Physics, Royal Surrey NHS Foundation Trust, Guildford GU2 7XX, UK; 4 St Luke’s Cancer Centre, Royal Surrey NHS Foundation Trust, Guildford GU2 7XX, UK; 5 Department of Medical Physics and Biomedical Engineering, University College London, London WC1E 6BT, UK; 6 Department of Nuclear Medicine, Christian Medical College Vellore, Vellore 632004, Tamil Nadu, India

**Keywords:** radiomics, reproducibility, repeatability, validation, lung cancer, head and neck cancer, CT imaging

## Abstract

Radiomics involves the extraction of information from medical images that are not visible to the human eye. There is evidence that these features can be used for treatment stratification and outcome prediction. However, there is much discussion about the reproducibility of results between different studies. This paper studies the reproducibility of CT texture features used in radiomics, comparing two feature extraction implementations, namely the MATLAB toolkit and Pyradiomics, when applied to independent datasets of CT scans of patients: (i) the open access RIDER dataset containing a set of repeat CT scans taken 15 min apart for 31 patients (RIDER Scan 1 and Scan 2, respectively) treated for lung cancer; and (ii) the open access HN1 dataset containing 137 patients treated for head and neck cancer. Gross tumor volume (GTV), manually outlined by an experienced observer available on both datasets, was used. The 43 common radiomics features available in MATLAB and Pyradiomics were calculated using two intensity-level quantization methods with and without an intensity threshold. Cases were ranked for each feature for all combinations of quantization parameters, and the Spearman’s rank coefficient, ***rs***, calculated. Reproducibility was defined when a highly correlated feature in the RIDER dataset also correlated highly in the HN1 dataset, and vice versa. A total of 29 out of the 43 reported stable features were found to be highly reproducible between MATLAB and Pyradiomics implementations, having a consistently high correlation in rank ordering for RIDER Scan 1 and RIDER Scan 2 (***rs*** > 0.8). 18/43 reported features were common in the RIDER and HN1 datasets, suggesting they may be agnostic to disease site. Useful radiomics features should be selected based on reproducibility. This study identified a set of features that meet this requirement and validated the methodology for evaluating reproducibility between datasets.

## 1 Introduction

There is growing evidence that standard-of-care medical images obtained from modalities such as CT, MRI, and PET contain more information than is visible to the human eye [1]. The high-throughput extraction and processing of the underlying information from radiological images is known as “radiomics”. The quantitative data obtained (imaging biomarkers) could potentially be used alongside the current gold standard of tumor evaluation and staging tools, including TNM staging [2], to aid clinical decision making such as personalized treatment planning.

The predictive power of radiomic features is dependent on having a large set of data. However, due to the nature of medical images, the size of the studies is often relatively small and based on a single dataset, restricting the impact of the results. To find candidates for reproducible biomarkers from the hundreds of features available from first, second, and higher-order statistical features of images, it is necessary for researchers to validate the results published by other groups [3]. This should be carried out using a separate dataset from the original study and considered a retrospective investigation. However, at least 50% of published studies have been described as poorly reported with incomplete methodologies and results for successful validation when an analysis of biomedical research was performed by Chalmers and Glasziou [3]. The precise cause of this serious lack of reproducibility in validation is unclear. The lack of standards for validating results, incomplete reporting of methodologies and results, and unrecognized confounding variables in the dataset used could all be to blame.

A recent systematic review of full-text articles in PubMed published in 2018 primarily addressed non-small cell lung cancer (NSCLC) and oropharyngeal cancer [4] and found only 7 out of 41 studies reported every methodology used in image acquisition, preprocessing, and feature extraction in detail. Out of 21 studies on NSCLC, 4 studies using CT images [5–8] and 1 study using PET (Positron Emission Tomography) images reported every methodologic aspect. The results identified the sensitivity of radiomic features in terms of repeatability and reproducibility to processing details such as the settings used in image acquisition, the image reconstruction algorithm, image preprocessing, and the software used to extract radiomic features. First-order features were reported to be more reproducible than shape metrics and texture features.

Our previously published study [9] analyzed radiomic features extracted from the CT component of PET/CT scans of patients with NSCLC, treated at the Royal Surrey NHS Foundation Trust (RSFT). The radiomics features were calculated using the toolkit of Vallières et al. [10], Which is available in the MATLAB package. This study found that a set of radiomics features were stable to settings used in image acquisition and reconstruction algorithms used in different scanner models. Features were also stable to variations in tumor delineation. However, features were sensitive to intensity quantization parameters, including (i) the number of intensity levels, (ii) the method of quantization to select the intensity levels, and (iii) the use of an intensity threshold around the tumor or organ being analyzed. These results show that different parameter choices in different datasets may help explain the results in the mentioned review papers [4,5,11]. Therefore, a question is: would these features be successfully reproduced and validated under different conditions, such as with a different lung cancer dataset, a different disease site, or using a different implementation of radiomics feature extraction?

## 2 Materials and Methods

The purpose of this paper is to investigate the generalizability of the findings from the initial study [9] and if a common set of CT radiomics features is stable. This was achieved first by evaluating which radiomics features are stable from the originally used 43 features of the Vallières toolkit, for a publicly available lung cancer dataset: the Reference Image Database to Evaluate Therapy Response (RIDER) [12]. As Pyradiomics [1] is one of the most used radiomics toolkits and provides the 43 features of the Vallières [10] plus 59 other original features, the RIDER dataset was also evaluated using Pyradiomics [13], and the results of the two toolkits were compared to study generalizability across radiomics implementations plus the extra features from Pyradiomics. As with the original study, this was carried out using the full intensity range in the images and thresholding to analyze the tumor intensity region only. To explore the applicability to other disease sites, a head and neck dataset was analyzed using the MATLAB toolkit and Pyradiomics. The dataset used was the HN1 dataset made publicly available in the Cancer Imaging Archive [1]. The stable features of HN1 were compared with those of RIDER.

### 2.1 Imaging Datasets

Two publicly available datasets were used in this study.

#### 2.1.1 RIDER Dataset

The RIDER dataset consists of non-contrast enhanced PET/CT images from pathologically confirmed NSCLC patients scanned at the Memorial Sloan-Kettering Cancer Center, New York, United States [12]. There were 31 patients in total, and they received conventional radiotherapy. Each patient had a repeat scan 15 min after the first scan, using the same scanner and imaging protocol. These are referred to as RIDER Scan 1 and RIDER Scan 2. The image datasets were acquired using two scanner types: GE LightSpeed RT16 and GE VCT. Each CT image size was 512 by 512 pixels, with pixel sizes ranging from 0.58 mm by 0.58 mm to 0.87 mm by 0.87 mm and a slice thickness of 1.25 mm.

#### 2.1.2 HN1 dataset

The HN1 dataset contains PET/CT images of 137 head and neck patients with squamous cell carcinoma. The patients were treated with definitive radiotherapy or concurrent chemoradiation. All patients underwent a treatment planning free-breathing 18F FDG-PET-CT scan (Biograph, SOMATOM Sensation-16 with an ECAT ACCEL PET scanner; Siemens, Erlangen, Germany), 45 min after uptake. A spiral CT (3 mm slice thickness) was performed, covering the complete thoracic region. Slice thickness: 1.5–3.0 mm; in-plane resolution: 0.9 × 0.98 mm^2^ to 1.09 × 1.09 mm^2^. The data also includes gross tumor volume (GTV) delineation by an experienced radiologist and a radiotherapy structure set. Further details are given here [1].

### 2.2 Texture Features Analyzed

To mimic the methodology of our previous study, the MATLAB texture analysis toolkit of Vallières et al. [13] was used to extract 43 standard features from the CT-defined GTV for the RIDER dataset. Three were first-order features, and 9 were from the gray level co-occurrence matrix (GLCM), the 13 gray level run length matrix (GLRLM), the 13 gray level size zone matrix (GLSZM) and the 5 neighboring gray tone difference matrixes (NGTDM). A full list of the features and equations defining them used for the 43 radiomics features can be found in the literature [10]. Using Pyradiomics a total of 103 features were extracted from the segmented GTV. These included: 17 first-order, 13 shape, 14 gray-level dependence matrix (GLDM), 22 GLCM, 16 GLRLM, 16 gray - GLSZM, and 5 NGTDM features.

### 2.3 Experimental Set-up and Statistical Analysis

The 43 features from the MATLAB toolkit were generated for both RIDER Scan 1 and Scan 2. Secondly, the results were also compared with and without an intensity threshold applied to the CT scan. The threshold used was −200 to 300 HU, as in our previous study [9]. Thirdly, stability was measured by comparing the global uniform quantizer (GUQ—with the same quantizer applied to each scan) and the individual uniform quantizer (IUQ—with the quantizer optimized for each scan). All these were uniform quantizers that quantized the intensity range of each GTV into equal width bins.

The same features were generated for the two RIDER scans using Pyradiomics with the same thresholding, and Fixed Bin Width (equivalent to Global Uniform quantizer) and Fixed Bin Count (equivalent to Individual Uniform Quantizer).

Results were compared between the MATLAB and Pyradiomics implementations and between the two RIDER scans, with and without intensity thresholds. No outcome information was available; hence, validation of the features was based on assessing the reproducibility of the rank ordering using each feature with changes to the quantization parameters for all datasets. Changes in the rank ordering indicate low reproducibility, leading to unreproducible predictions of biomarkers. In addition, the stability of the other 59 features available in Pyradiomics was also studied. Although these cannot be used to comment on the consistency of the MATLAB toolkit, it is instructive to determine if they are candidate stable features.

A feature was considered reproducible if it produced the same rank ordering for the cohort regardless of the quantization parameters. Spearman’s rank correlation, ***rs***, was used to measure the rank ordering quantized using IUQ against GUQ at 128 intensity levels used as a reference with and without intensity thresholding, for all datasets. The rank ordering quantized with GUQ at 128 intensity levels as a reference was used as the standard comparator as it was found to be the most stable quantization combination [9]. Validation was considered successful if a feature that expressed high or low correlation in the MATLAB toolkit also expressed high or low correlation in Pyradiomics.

In the comparison of stable features between arms of the study in the results, e.g., between radiomics toolkits and disease sites, Venn diagrams are used to illustrate which features show promise as stable features in multiple arms.

## 3 Results

Table 1 lists all features that were reproducible, with high correlation, for RIDER Scans 1 and 2 based on the Spearman’s correlation coefficient with threshold (blue) and without threshold (red) using the two quantizers GUQ and IUQ. Features with ***rs*** ≥ 0.75 were classified as highly correlated. This was a subjective decision as no published guidance was available. A total of 34 features were found to be reproducible: 29 and 21 with and without intensity threshold applied, respectively, and features were found to be reproducible for all data. This suggests that including intensity threshold around the region of interest as a pre-processing step tends to increase the stability of some radiomics features.

**Figure 1:**
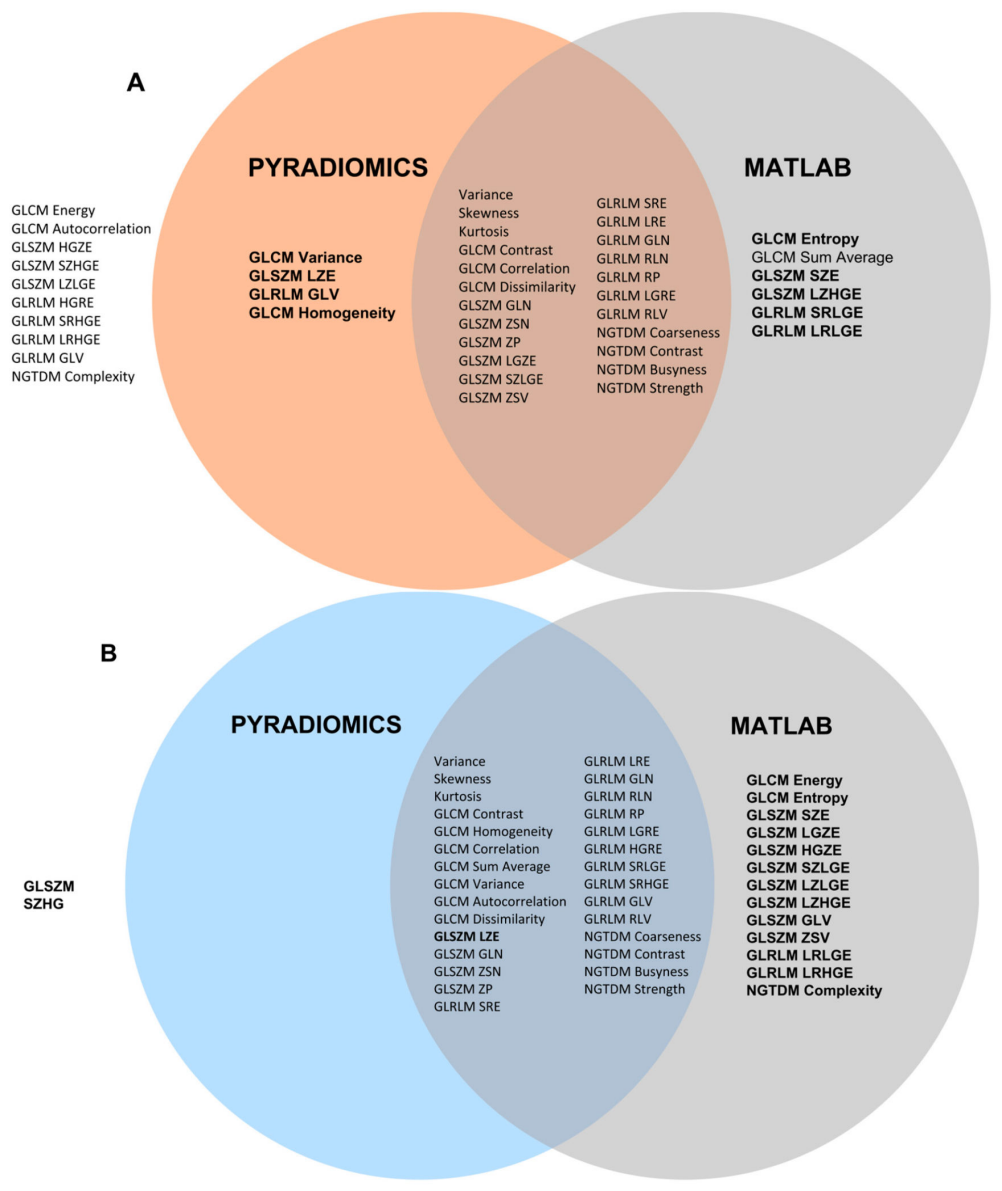
Venn diagrams with a visual representation of features that are reproducible between MATLAB and Pyradiomics feature extraction toolkits for GUQ versus IUQ with 128 quantizer levels (Fixed Bin Width in Pyradiomics). (**A**) without threshold; (**B**) with threshold applied. Reproducibility is measured by an ***rs*** value greater than or equal to 0.8. Features highlighted in bold had ***rs*** value greater than 0.9.

Figure 1 shows a Venn diagram plotting the overlap in highly correlated features quantized with GUQ versus IUQ for 43 features that are commonly available in the MATLAB toolkit and Pyradiomics. Panels A and B plot the correlation of rank ordering quantized between GUQ and IUQ without and with the use of an intensity threshold. The correlation value for most shape, first order, GLCM, GLRLM, GLZSM, and NGTDM texture features were high (>0.8) for the RIDER Scan 1, with 9 and 14 features with ***rs*** > 0.9 without and with intensity threshold. The number of features that overlapped between the MATLAB toolkit and Pyradiomics was higher when the intensity threshold was applied. A similar trend was seen for RIDER scan 2 data.

**Figure 2:**
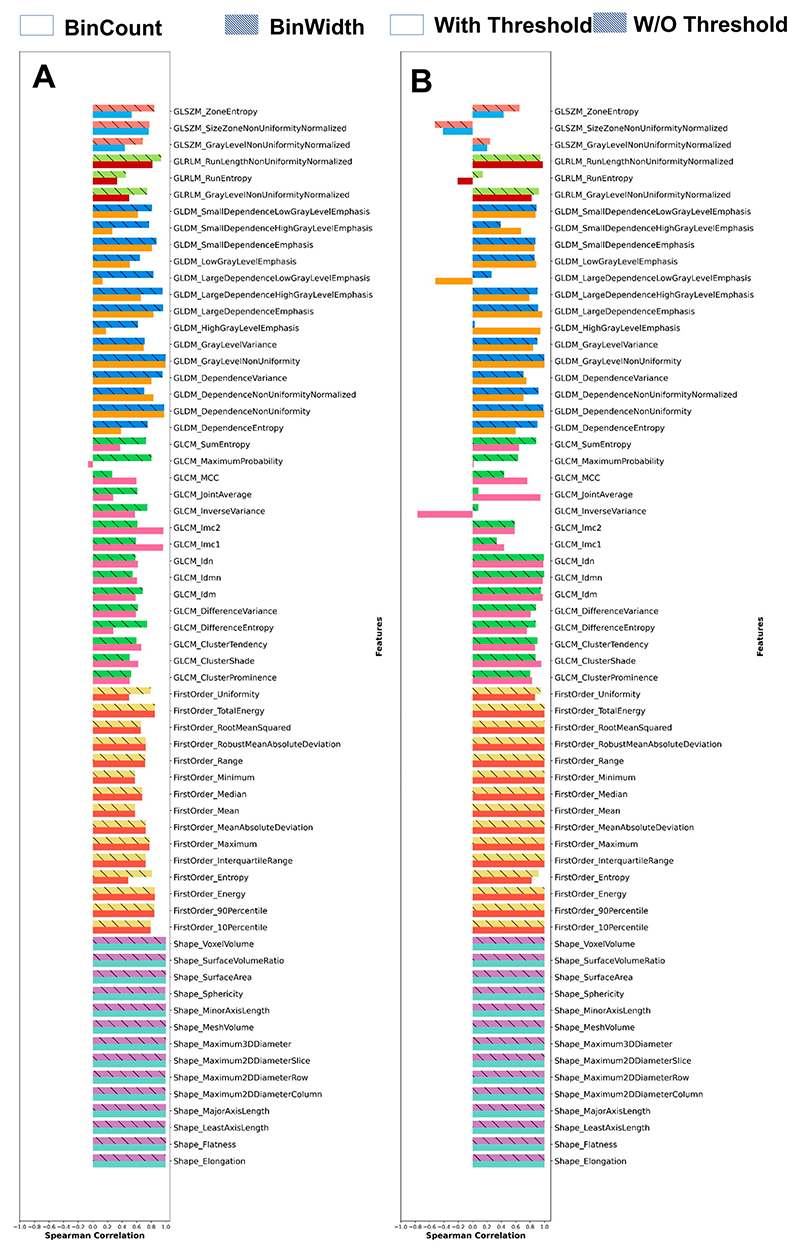
The Spearman correlation ranks of features extracted using (**A**) threshold versus no threshold and comparing the impact of the quantization techniques (GUQ (bin count) vs. IUQ (bin width)) and (**B**) correlation between quantization methods (GUQ and IUQ) and comparing the impact of thresholding. Each color-paired block represents a separate feature class.

Figure 2 presents the results for features that were uniquely available in Pyradiomics. Figure 2A shows the correlation of rank order between features extracted with and without an intensity threshold with the use of GUQ and IUQ quantization. Similarly, Figure 2B shows the correlation of ranks quantized between GUQ and IUQ with and without intensity threshold being applied. Shape features were found to be invariant to intensity thresholds or quantization techniques. For all other classes, the features showed more variance based on the application of an intensity threshold and less so on the quantization used. A few features showed negative correlation with the choice of quantization used, namely GLCM Inverse Variance, GLDM Large Dependence Low Gray Level Variance, GLRLM Run Entropy, and GLZSM Size Zone Nonuniformity Normalized. This negative correlation was seen only in the GLCM Maximum Probability when an intensity threshold was applied.

**Figure 3:**
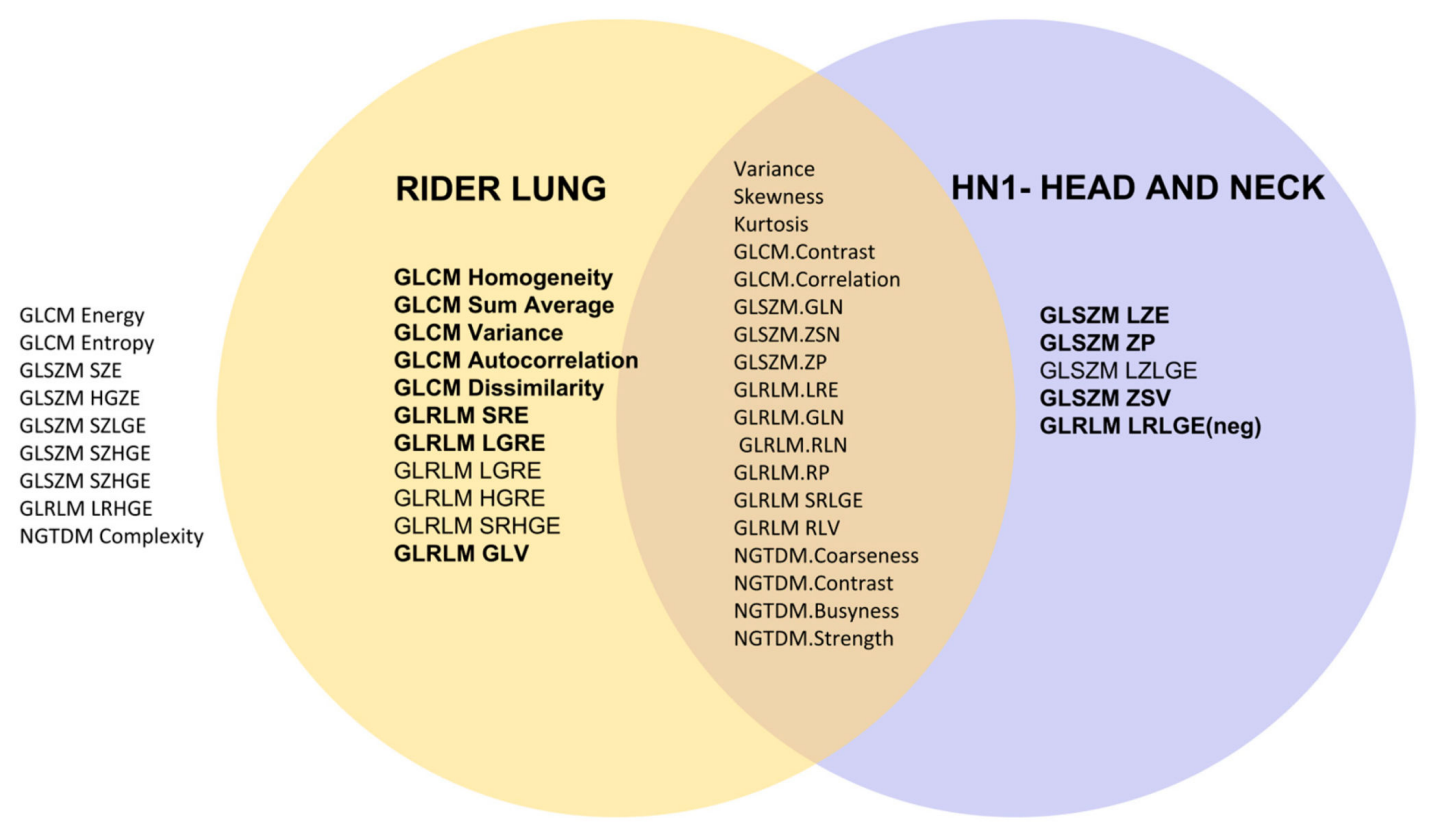
Venn diagram with a visual representation of features that are reproducible between RIDER Lung 1 and the Head and Neck (HN1) dataset and with an intensity threshold applied. Reproducibility is measured by an ***rs*** value greater than or equal to 0.8. Features highlighted in bold had an rs value greater than 0.9.

Figure 3 plots a Venn diagram with the overlap in the highly correlated features quantized between GUQ and IUQ and with an intensity threshold applied for datasets RIDER Lung 1 and Head and Neck HN1.The features were extracted using Pyradiomics; 18/43 features were agnostic of the disease site and 4/5 from HN1 and 8/11 from RIDER lung had rank correlations above 0.9, respectively.

## 4 Discussion

The purpose of this study was to verify the robustness of the methods reported earlier for improving the stability of radiomic features. The study used two different datasets with different pixel sizes acquired in a different center using different scanners and protocols, one for lung cancer and the other for head and neck cancer, both available publicly, while additionally testing for reproducibility and stability in the implementation of radiomic features. High levels of correlation were achieved for more than half of the features for both MATLAB and Pyradiomics implementations for the RIDER lung data, and more than one-third of the features were stable for lung and head and neck datasets, suggesting that some features are agnostic to the disease site and generally robust.

There are several characteristics of imaging systems that are relevant to their use in healthcare. These include pixel size and spatial resolution, acquisition parameters (such as kVp in x-rays and imaging sequence in MRI), tomographic reconstruction parameters, patient positioning, and when the image is taken on the patient pathway. These are all important parameters at the start of the process and are determined before radiomics analysis is performed. One of the biggest challenges in radiomics is the low reproducibility of the results from various studies [4,11]. Some of the possible causes for the low reproducibility include pre-existing differences in the dataset used, for example, different acquisition parameters [14,15], reconstruction methods [16,17], pixel sizes [18] and slice thickness; low reproducibility of features due to variations in quantization parameters; and low repeatability of the features [14]. Other considerations include the preprocessing of the imaging data [19]. For instance, Mottola et al. studied the effects of image resampling and showed that different resampling approaches produced very different error metrics, with Lanczos interpolation performing substantially better than simple linear interpolation [20]. Cui and Yin have detailed the impact that image quality has on radiomic applications and summarized the minimum image quality requirements and recommendations for reducing the impact of image quality on the reproducibility of radiomic studies [11]. Broadly, for radiomics studies, it is important that all parameter choices are documented and reported, and more specifically, it is good practice that imaging parameters are kept as consistent as possible. Binsheng Zhao suggests quantitative methods/metrics to help determine image quality and/or similarity to recognize comparable images that can be used interchangeably or to decide whether an image’s quality is adequate for computing radiomics features [21]. Often, radiomic features identified as predictive are based on small datasets, may be biased toward the specific dataset, and have limited predictive power on another dataset. For other sources of variability affecting radiomics models, readers are directed to some of these studies [19,21,22].

The aim of many radiomics studies, including those by our group, is to predict an outcome such as response to treatment or disease-free survival using one or multiple features referred to as biomarkers. Predictions are often performed using statistical approaches, including Kaplan-Meier analysis based on a single feature of the data at a time [23] and machine learning approaches with multiple features from a large set of features up to hundreds [1,24]. Outcome prediction accuracy is heavily reliant on having highly reproducible features. For instance, the widely used Kaplan-Meier analysis method involves ordering the dataset based on a feature and dichotomizing it into two sets for prediction. It is vital for the rank ordering to be consistent, as changes in the rank order may change the dichotomization and hence results in Kaplan-Meier studies, leading to low reproducibility and low predictive power. In our previous work [9], we reported a methodology to evaluate the rank order of the features and have shown that some radiomics features are reproducible across different scanner models, acquisition parameters, reconstruction methods, and modest variations in slice thickness, provided pixel sizes are resampled to a fixed standard. It was identified that feature reproducibility was highly sensitive to the choice of quantization parameters. This study has successfully validated our previous results [9] and reproduced the changes in radiomics features using different quantization parameters, suggesting the methodology used for the study is robust, even when using a different radiomics feature extraction implementation [3]. These results highlight the importance of reporting the detailed methodology used. Based on studies in the literature [11,14,25] and our own results, we recommend excluding unreproducible features from analysis to reduce dimensionality and computational burden. To improve further studies have suggested that deep learning could be considered to improve the image quality of the CT images [26], which may lead to reproducible radiomics features [27]. This will need to be explored further in future studies. In this era of deep learning, Chung et al. have raised a question for further thought for the radiology and quantitative imaging communities: have we already lost a lot of information available when we choose to reconstruct images for visual interpretation? [28].

There are some limitations to this study. Pyradiomics does not comply completely with all the recommendations of the Imaging Biomarker Standardization Initiative (IBSI) [27], for example, the quantization parameters. Although care was taken to keep the suggested stability parameters as close as possible, their implementation would have affected the present study. Hence, IBSI compliance is strongly recommended to allow better reproduction and validation of the treatment results externally [29]. The goal of all radiomics studies is to predict clinically relevant properties and/or disease outcomes, such as disease recurrence or survival. The study has only focused on the reproducibility of the features; however, the reproducibility of a feature does not automatically imply that it is clinically informative. The next stage in evaluating this methodology will be to apply it to the modeling of outcomes.

## 5 Conclusions

Radiomics features reported as stable were analyzed for reproducibility using the RIDER lung dataset, with 29 of 43 features found to be reproducible to changes in the feature extraction toolkits when intensity threshold was applied, maintaining stable rank ordering (***rs*** > 0.8), and are recommended for use for biomarker analysis. We found that 18/43 reported features were common in the RIDER and HN1 datasets, suggesting they may be agnostic to disease site. Useful radiomics features should be selected based on reproducibility. This study identified a set of features that meet this requirement and validated the methodology for evaluating reproducibility between datasets.

## Supplementary Material

Appendix

## Figures and Tables

**Table 1:** List of radiomics features with reproducibility with threshold and without threshold shown in blue and black for RIDER Scans 1 and 2, respectively, in columns 2, 3, 4 and 5 for MATLAB, columns 6, 7, 8, and 9 for Pyradiomics. Spearman correlation coefficient values ≤0.75 is shown in red. Columns 10 and 11 represent the reproducible features across both scans and implementations; with threshold, are shown in orange tick and without threshold in purple tick, respectively; across all data is shown by a green tick in column 12. Colours are explained in the text.

Radiomics Feature	Matlab				Pyradiomics				Across Scans 1 & 2 and Feature Extraction Implementations		All Data
	Scan 1	Scan 2	Scan 1	Scan 2	Scan 1	Scan 2	Scan 1	Scan 2	Threshold	W/o Threshold	
	Threshold		W/o Threshold		Threshold		W/o Threshold				
Variance	1.00	1.00	1.00	0.99	1.00	1.00	1.00	1.00	**✓**	**✓**	**✓**
Skewness	1.00	1.00	1.00	0.99	1.00	1.00	1.00	1.00	**✓**	**✓**	**✓**
Kurtosis	1.00	1.00	1.00	0.98	1.00	1.00	1.00	1.00	**✓**	**✓**	**✓**
GLCM Energy	0.95	0.96	0.73	0.83	0.16	0.00	0.71	0.00			
GLCM Contrast	1.00	1.00	0.80	0.81	0.85	0.95	0.91	0.95	**✓**	**✓**	**✓**
GLCM Entropy	0.98	0.97	0.79	0.84	–0.03	–0.10	0.50	–0.10			
GLCM Homogeneity	0.96	0.98	0.69	0.75	0.93	0.97	0.94	0.97	**✓**		
GLCM Correlation	1.00	1.00	1.00	0.98	0.92	0.98	1.00	0.98	**✓**	**✓**	**✓**
GLCM Sum Average	1.00	1.00	0.18	0.27	0.95	0.90	0.08	0.90	**✓**		
GLCM Variance	1.00	1.00	0.80	0.81	0.87	0.98	0.92	0.98	**✓**	**✓**	**✓**
GLCM Autocorrelation	1.00	1.00	0.15	0.23	0.94	0.88	0.06	0.88	**✓**		
GLCM Dissimilarity	1.00	1.00	0.80	0.82	0.89	0.97	0.94	0.97	**✓**	**✓**	**✓**
GLSZM SZE	0.80	0.77	0.83	0.84	–0.42	–0.17	–0.54	–0.17			
GLSZM LZE	0.94	0.90	0.75	0.82	0.76	0.74	0.88	0.74	**✓**		
GLSZM GLN	0.98	0.98	0.88	0.86	0.98	0.99	0.97	0.99	**✓**	**✓**	**✓**
GLSZM ZSN	0.81	0.77	0.83	0.84	0.99	0.98	0.96	0.98	**✓**	**✓**	**✓**
GLSZM ZP	0.87	0.88	0.78	0.86	0.79	0.79	0.79	0.79	**✓**	**✓**	**✓**
GLSZM LGZE	0.99	0.99	0.84	0.81	0.39	0.23	0.79	0.23			
GLSZM HGZE	0.97	0.98	0.17	0.21	0.22	0.34	–0.07	0.34			
GLSZM SZLGE	0.99	0.99	0.87	0.81	–0.06	0.14	0.78	0.14			
GLSZM SZHGE	0.74	0.79	0.06	0.05	0.24	0.35	–0.21	0.35			
GLSZM LZLGE	0.99	1.00	0.55	0.43	–0.72	–0.70	0.34	–0.70			
GLSZM LZHGE	0.92	0.91	0.93	0.84	0.49	0.57	0.87	0.57			
GLSZM GLV	0.98	0.99	0.65	0.71	0.01	–0.12	–0.22	–0.12			
GLSZM ZSV	0.98	0.93	0.81	0.74	0.73	0.73	0.87	0.73			
GLRLM SRE	0.97	0.97	0.75	0.80	0.76	0.95	0.94	0.95	**✓**	**✓**	
GLRLM LRE	0.97	0.99	0.75	0.79	0.97	0.96	0.90	0.96	**✓**	**✓**	
GLRLM GLN	0.93	0.96	0.82	0.90	0.99	0.99	0.99	0.99	**✓**	**✓**	**✓**
GLRLM RLN	0.97	0.97	0.75	0.80	0.99	0.99	0.99	0.99	**✓**	**✓**	
GLRLM RP	0.97	0.98	0.75	0.80	0.98	0.97	0.92	0.97	**✓**	**✓**	
GLRLM LGRE	1.00	1.00	0.87	0.86	0.86	0.88	0.88	0.88	**✓**	**✓**	**✓**
GLRLM HGRE	1.00	1.00	0.13	0.19	0.96	0.83	0.01	0.83	**✓**		
GLRLM SRLGE	1.00	1.00	0.88	0.87	0.93	0.95	0.86	0.95	**✓**	**✓**	**✓**
GLRLM SRHGE	0.99	1.00	0.15	0.18	0.83	0.69	–0.03	0.69	**✓**		
GLRLM LRLGE	1.00	1.00	0.88	0.85	–0.41	–0.36	0.45	–0.36			
GLRLM LRHGE	0.99	0.99	0.21	0.39	0.18	0.30	0.33	0.30			
GLRLM GLV	1.00	0.99	0.63	0.76	0.76	0.87	0.85	0.87	**✓**		
GLRLM RLV	0.94	0.95	0.82	0.73	0.96	0.95	0.91	0.95	**✓**		
NGTDM Coarseness	0.99	0.99	1.00	0.96	1.00	1.00	1.00	1.00	**✓**	**✓**	**✓**
NGTDM Contrast	1.00	1.00	0.96	0.96	0.97	0.97	0.98	0.97	**✓**	**✓**	**✓**
NGTDM Busyness	0.99	0.99	0.94	0.93	1.00	0.99	0.97	0.99	**✓**	**✓**	**✓**
NGTDM Complexity	1.00	1.00	–0.30	–0.20	0.71	0.73	–0.10	0.73			
NGTDM Strength	1.00	1.00	0.93	0.92	1.00	1.00	0.96	1.00	**✓**	**✓**	**✓**

## Data Availability

This study used two publicly available datasets: RIDER and HN1. The RIDER dataset is available from the Cancer Imaging Archive: https://wiki.cancerimagingarchive.net/display/Public/RIDER+Lung+CT (accessed on 17 June 2023). Details of HN1 are given in reference [1]. The MATLAB code used is from the toolbox of Vallières: https://github.com/mvallieres/radiomics/tree/master/TextureToolbox (accessed on 17 June 2023). The functions used were: prepareVolume.m, equalQuantization.m, getGLCM.m, getGLSZM.m, getGLRLM.m, getNGTDM.m, and getGlobalTextures.m. The MATLAB user code is given in Appendix A. The Pyradiomics parameter file used for the feature extraction is given in Appendix B.

## Abbreviations

GLCM: Gray-Level Co-Occurrence Matrix
GLDM: Gray-Level Dependence Matrix
GLRLM: Gray Level Run Length Matrix
GLSZM: Grey Level Size Zone Matrix
GTV: Gross Tumor Volume
GUQ: Global Uniform Quantizer
ICC: Interclass Correlation
IUQ: Individual Uniform Quantizer
NGTDM: Neighboring Gray Tone Difference Matrix
RIDER: Reference Image Database to Evaluate Therapy Response
***rs***: Spearman’s rank correlation
