# Supplementary material for: Reproducibility in Radiomics: A Comparison of Feature Extraction Methods and Two Independent Datasets
Source: Appl Sci (Basel). Author manuscript; Available in PMC 2024 May 9. (PMC7615943; doi:10.3390/app13127291)
Supplement: Appendix [file EMS195909-supplement-Appendix.pdf]

Appendix A. MATLAB User File

```
foldername = 'folder with GTVseg';
cd 'to your working directory';
for ns = 1:length(s)
filename = ['GTVsegmentation_pt' num2str(s(ns)) '.mat'];
files = load(fullfile(foldername,filename));
names=fieldnames(files);
for int= 1:size(names,1)
volume = files.(names{int,1});
```

```
end
mask=volume;
mask(mask>0)=1;

% thresholding
volume_low = 800 <= volume;
volume_high = volume <= 1300;
volume = volume.*volume_low;
volume = volume.*volume_high;
texture = zeros(1,43);

% Specify the Quantization level
for Qsize = 128
% choose quantiser
% https://github.com/mvallieres/radiomics/blob/master/TextureToolbox/Pre-processing
/prepareVolume.m
GUQ
[ROIonly,levels] = prepareVolume(volume,mask,'Other','pixelW','sliceS',1,'pixelW',
'Matrix','UniformRange',Qsize);
% IUQ
[ROIonly,levels] = prepareVolume(volume,mask,'Other','pixelW','sliceS',1,'pixelW',
'Matrix','Uniform',Qsize);

ROIOnly = ROIonly;

% generate texture features using toolbox
% https://github.com/mvallieres/radiomics/tree/master/TextureToolbox/GLCM/getGLCM.m
% implements quanitiser from toolbox
% https://github.com/mvallieres/radiomics/blob/master/TextureToolbox
/Pre-processing/Quantization/equalQuantization.m
[GLCM] = getGLCM(ROIonly,levels);
[textures] = getGLCMtextures(GLCM);
texture(Qsize,1:9) = [textures.Energy textures.Contrast textures.Entropy textures.
Homogeneity textures.Correlation textures.SumAverage textures.Variance textures.
Dissimilarity textures.AutoCorrelation];

% https://github.com/mvallieres/radiomics/tree/master/TextureToolbox/GLSZM/
getGLSZM.m
[GLSZM] = getGLSZM(ROIOnly,levels);
[textures] = getGLSZMtextures(GLSZM);
texture(Qsize,10:22) = [textures.SZE textures.LZE textures.GLN textures.ZSN textures.ZP
textures.LGZE textures.HGZE textures.SZLGE textures.SZHGE textures.LZLGE
textures.LZHGE textures.GLV textures.ZSV];

% https://github.com/mvallieres/radiomics/tree/master/TextureToolbox/GLRLM/
getGLRLM.m
[GLRLM] = getGLRLM(ROIonly,levels);
[textures] = getGLRLMtextures(GLRLM);
texture(Qsize,23:35) = [textures.SRE textures.LRE textures.GLN textures.RLN textures.RP
textures.LGRE textures.HGRE textures.SRLGE textures.SRHGE textures.LRLGE
textures.LRHGE textures.GLV textures.RLV];

% https://github.com/mvallieres/radiomics/tree/master/TextureToolbox/NGTDM/
getNGTDM.m
```

```
[NGTDM,countValid] = getNGTDM(ROIOnly,levels);
[textures] = getNGTDMtextures(NGTDM,countValid);
texture(Qsize,36:40) = [textures.Coarseness textures.Contrast textures.Busyness textures.
Complexity textures.Strength];

% https://github.com/mvallieres/radiomics/tree/master/TextureToolbox/Global/
getGlobalTextures.m
[textures] = getGlobalTextures(ROIonly,Qsize);
texture(Qsize,41:43) = [textures.Variance textures.Skewness textures.Kurtosis];

% save after each quantisation level
save(([′texture_pt′ num2str(s(ns)) ′.mat′]),′-v7.3′,′texture′);
disp([′pt′ num2str(s(ns)) ′ n′ num2str(Qsize)])

end
```

Appendix B. Pyradiomics Parameter File

```
Extracted using PyRadiomics version: 2.1.0
imageType:
Original:
binCount: 128
featureClass:
glcm:
- ′JointEnergy′
- ′Contrast′
- ′JointEntropy′
- ′Id′
- ′Correlation′
- ′SumAverage′
- ′SumSquares′
- ′DifferenceAverage′
- ′Autocorrelation′
glszm:
- ′SmallAreaEmphasis′
- ′LargeAreaEmphasis′
- ′GrayLevelNonUniformity′
- ′SizeZoneNonUniformity′
- ′ZonePercentage′
- ′LowGrayLevelZoneEmphasis′
- ′HighGrayLevelZoneEmphasis′
- ′SmallAreaLowGrayLevelEmphasis′
- ′SmallAreaHighGrayLevelEmphasis′
- ′LargeAreaLowGrayLevelEmphasis′
- ′LargeAreaHighGrayLevelEmphasis′
- ′GrayLevelVariance′
- ′ZoneVariance′
glrlm:
- ′ShortRunEmphasis′
- ′LongRunEmphasis′
- ′GrayLevelNonUniformity′
- ′RunLengthNonUniformity′
- ′RunPercentage′
- ′LowGrayLevelRunEmphasis′
- ′HighGrayLevelRunEmphasis′
```

- 'ShortRunLowGrayLevelEmphasis'
- 'ShortRunHighGrayLevelEmphasis'
- 'LongRunLowGrayLevelEmphasis'
- 'LongRunHighGrayLevelEmphasis'
- 'GrayLevelVariance'
- 'RunVariance'

ngtdm:
- 'Coarseness'
- 'Contrast'
- 'Busyness'
- 'Complexity'
- 'Strength'

firstorder: # Remove Total Energy, correlated to Energy (due to resampling enabled)
- 'Variance'
- 'Skewness'
- 'Kurtosis'

setting:
Resampling:
interpolator: 'sitkLinear'
resampledPixelSpacing: [0.98, 0.98, 0]
resegmentRange: [−200, 300]
resegmentMode: absolute
Misc:
label: 1
